# 

**Published:** 1883-10

**Authors:** 


### BOOKS AND PAMPHLETS RECEIVED.

STUDIES FROM THE BIOLOGICAL LABORATORY, edited by Newell Martin, M.A.D.Sc., M.D., and W. K. Brooks, Ph.D. Vol. II., No. 3. Johns Hopkins University, Baltimore. July, 1883.

OBSERVATIONS ON THE MANAGEMENT OF ENTERIC FEVER ACCORDING TO A PLAN BASED UPON THE SO-CALLED SPECIFIC TREATMENT, by James C. Wilson, M.D. Philadelphia, 1883.

ANNUAL ANNOUNCEMENT OF THE COLUMBIA VETERINARY COLLEGE. 1883—1884.

ANNUAL ANNOUNCEMENT OF THE AMERICAN VETERINARY COLLEGE. 1883—1884.

ANNUAL ANNOUNCEMENT OF THE CHICAGO VETERINARY COLLEGE. 1883—1884.

JOHNS HOPKINS UNIVERSITY CIRCULARS.

ANNALS ET BULLETIN DE LA SOCIÉTÉ DE MEDICINE DE GAND.

THE POLYCLINIC, Published by P. Blakiston, Son & Co., Philadelphia.

JOURNALS.—La Clinica Veterinaria Milano.

The Veterinarian, London; The Veterinary Journal, London; Der Zoologische Garten, Frankfort; Schweizerisches Archiv fur Thierheilkunde, und Thierzucht, Bern. Archiv fur Wissenschaftliche und Practische Thierheilkunde, Berlin; Journal de la Société contre L'Abus du Tabac, Paris; Tidskrift for Veterinar-Medicine och Husdjursskotsel utgifven af C. A. Lindqvist, Stockholm; Giornale di Anatomia, Fisiologia e Patologia, degli Animali, Pisa; Il Medico Veterinario, Torino; Kansas City Review of Science and Industry; Chicago Medical Journal and Examiner; The Medical Record, New York; The College and Clinical Record, Philadelphia; Annals of Anatomy and Surgery, Brooklyn; New England Medical Monthly, Sandy Hook; Chicago Medical Review: Virginia Medical Monthly, Richmond; Nashville Journal of Medicine and Surgery; The Southern Clinic, Richmond; The Western Medical Reporter, Chicago; Journal of Cutaneous and Venereal Diseases, New York; Denver Medical Times; The St. Joseph Medical Herald, San Francisco Western Lancet; The Weekly Medical Review, Chicago and St. Louis; The Blacksmith and Wheelwright, New York; National Live Stock Journal, Chicago; The Breeder's Gazette, Chicago; Cultivator and Country Gentleman, Albany; Indiana Farmer, Indianopolis; Truth, San Francisco; Weekly Drover's Journal, Chicago; The Chicago Tribune; The Medical Register, Philadelphia, Spirit of the Times, New York; United States Veterinary Journal Chicago; Horseshoer and Hardware Journal, Chicago.

### INDEX.

| | PAGE. |
|---|---|
| A bacillus or a fat crystal | 73 |
| Abnormal periods of gestation | 255 |
| Abortion | 204 |
|     epizootic cellulitis | 237 |
|     in cows | 109, 139 |
| About cattle liable to | 320 |
| A cat's attempt at suicide | 82 |
| Acknowledgement | 149 |
| A cow as is a cow | 83 |
| Actinomykosis | 183 |
| Adams, W. S.—castration statistics | 281 |
| Adenoma in a dog | 59 |
| Affinities, physiological, into the blood of animals | 8, 104 |
| A horse calls on a doctor | 182 |
| Air space in cow sheds | 256 |
| Animal forms, causes of changes in | 19, 94 |
| Anthrax in the hog different from common form | 251 |
| Arthritis, chronic rheumatic | 163 |
| Ascaris mystax in cats | 171 |
| Atavism and reproduction | 36, 113 |
| Attempts at Veterinary Colleges | 51 |
| | |
| Bacteria in syphilis | 182 |
| Balling of horses, a contrivance to prevent | 180 |
| Bassi, Prof. R.—cutaneous disease in the horse | 295 |
| Bates, E. S.—oleum gaultheria | 128 |
| Bequests to Dick Veterinary College | 330 |
| Berns, Dr.—bursal enlargement of the tendon of the flexor pedis perforans | 237 |
| Billings, Frank S.—the U. S. Government in its relation to contagious animal diseases and the veterinary profession | 263 |
|     a case of strychnine poisoning in a dog | 235 |
| Birds and trichina | 74 |
| Bitten by a squirrel | 180 |
| Blinkers | 327 |

Blood poisoning........................................................ 82
Books and pamphlets received..........................72, 165, 246, 319
Boston dog destroyer................................................ 327
Bowels in health and disease, the movements of..................... 176
Bower, Dr.—spinal meningitis........................................ 239
Bowler, G. W.—latent glanders in the horse.......................... 1
Bronchitis in birds................................................. 73
Bursal enlargement of the tendon of the flexor pedis perforans...... 237

Cancer of stomach................................................... 324
Cartilage, the reparative process in................................ 252
Carbolic acid in catarrh............................................ 62
Case department...........................................50, 150, 235, 313
Castration statistics............................................... 231
Cataract, remarkable cure of in a dog............................... 332
Cattle plague, the.................................................. 328
Cattle, the breeds of their diseases and treatment.................. 56
the treatment of imported........................................... 260
Chicken cholera..................................................... 320
Cholera in a horse.................................................. 244
Clover bloat........................................................ 327
Clement, A. W.—lesions in a case of immobilite...................... 150
Comparative physiology, tables in................................... 181
dietetics........................................................... 78
pathology, the progress of.......................................... 132
Cerebral congestion................................................. 262
Consumption and tuberculosis........................................ 253
Contagious animal diseases.......................................... 263
Contraction of the foot in the horse................................ 273
Correspondence.......................................................64, 234
Cow, softening of bones in the...................................... 74
Cranial capacity of the horse....................................... 75
Cruelty to animals.................................................. 256
Cutaneous disease in horse.......................................... 295
Cyst, removal of ; in pointer........................................ 316
Cystomy, supra-pubic................................................ 213

Dana, Charles L.—some recent contributions to the physiology of the nervous system of our domestic animals........................ 101
Dead animals, removal of............................................ 327
Delisser, G. P.—idopathic tétanus................................... 61
penetrating wound of abdomen........................................ 159
Dick, Miss.—the late................................................ 317
Digitaline, influence of............................................ 172
Diptheria........................................................... 260
of the bladder in a rabbit........................................... 173
Diseased meat in Glasgow............................................ 327

Dolphin, parasitic disease in a.... 74
Domestication of the horse.... 328
Donation to Veterinary College.... 81

Editorials....50, 132, 225, 306
Elephants, insane.... 261
sexual mania in.... 54
destroyed by prussic acid.... 326
Elephantiasis in a horse.... 74
Ely, Edward P.—a severe case of spongy iritis in a horse.... 59
Eye in lower animals, some diseases of....31,190
Experiments on animals with male fern.... 175
Exports and imports and quarantine of neat cattle.... 84

Farcy, acute in man.... 321
Fibro-myomata of the uterus of the sea lion.... 174
Filaria oculi occurring in both eyes of a horse.... 182
Flexible anatomical preparation of joints.... 77
Form of the pulse wave.... 177
Fowls, diseased.... 73
Frey, Mark L.—hepatitis in a dog.... 314
Fracture of the internal metatarsal.... 162
os inominata.... 164
femur in a puma.... 321
multiple comminuted, of the sesamoid bones.... 241
of os suffraginis.... 313
treatment of.... 140

Gastric juice of the horse's stomach.... 309
Germ theory, the.... 225
Glanders....306, 260
etiology of.... 171
in the horse.... 1
Goats and homœopathy.... 55
milk, the daily yield of.... 81

Hœmaturia associated with nephritis.... 161
Hawk, Dr.—pleuro-pneumonia contagiosa.... 243
Heath, A. S.- abortion.... 204
are there physiological affinities in the blood of each breed of animals....8, 104
Hepatitis in a dog.... 314
Heredity.... 73
History of veterinary medicine and surgery in the U. S., the early.... 42
Hock, principal diseases of.... 321
Hog cholera, the germ of.... 179
Homatropine, hydrobromate of.... 74

Horses for food.................................................. 256
family.................................................. 74
railways.................................................. 331
Hospital for animals.................................................. 181
Hydatids of the liver from a camel.................................................. 243
Hydrophobia, death from.................................................. 329
the prevention of.................................................. 178
a case treated successfully with aconite.................................................. 76

Irido-choroiditis.................................................. 234
Induced predisposition to anthrax.................................................. 80
Influence of animal food on the production of wool .................. 79
Inoculation of animals for the prevention of pleuro-pneumonia....... 130
monkeys with syphilis.................................................. 171
for pleuro-pneumonia.................................................. 308
Intelligence in animals.................................................. 182
Interesting to veterinary dissectors.................................................. 76
Intestinal calculi..................................................162, 242
disorder in bones.................................................. 77
obstruction.................................................. 174
Iritis in a horse.................................................. 59
Itch in the cat.................................................. 320

Jaundice, a case of.................................................. 240
Jennings, R.—the early history of veterinary medicine.............. 42
the teeth of the horse and their diseases.............. 121
Journalistic changes.................................................. 140

Keratitis in a dog.................................................. 242
Kidneys, acute parenchymatous degeneration of the.................. 87
in a mare.................................................. 156

Lameness..................................................215. 298
simple.................................................. 240
Legislation regarding neat cattle.................................................. 84
Lesion in a case of immobilite.................................................. 150
Lindsay, John.—treatment of chronic catarrh by carbolic acid........ 62
recurring cystic tumors of the supra-scapular region. 157
acute parenchymatous nephritis.................. 154
chorea in a horse.................................................. 244
Liver, cystic diseases of, in a pig.................................................. 313
Loss of cud.................................................. 181
Lovell, R.—fracture of os suffraginis.................................................. 313
traumatic or idiopathic tetanus.................................................. 151
Lowe, William Herbert.—cases.................................................. 315

Male fern........ 173
Maternal impressions........ 329
McLellan, E. A.—Lameness........ 215, 298
Multiple comminuted fracture of the sesamoid bones.. 241
Abortion epizootic cellulitis........ 237
Medicine as practised by animals........ 176
Meningitis, spinal........ 239
Menstruation, comparative physiology of........ 310, 284
Milk sickness........ 117
Misnomer explained, a........ 256
Mitchell Hubbard W.—Causes of change in animal forms........ 19
Atavism........ 113
Monkeys, diseases of........ 331
Montreal Veterinary College........ 167
Moore, William Oliver.—Keratitis in a dog........ 242
Some diseases of the eye in lower animals.. 31, 199
Ingrowing eyelashes........ 236
Moor tops for stable bedding........ 331

Navin, John N.—Atavism and reproduction........ 36
Nephritis, acute parenchymatous........ 154
Nervous diseases of pachyderms........ 75
Nervous system of our domestic animals, recent contributions to the physiology of........ 101
Newman, Dr.–Novel way of removing a foreign body from the œsophagous. 237
News and Miscellaney........ 81, 180, 254, 236

Obituary........ 67, 166, 247, 371
O'Leary, Cornelius M.—The extent to which animal forms undergo change........ 94
Oleum Gaultheria—oil of wintergreen........ 128
Orang-outang in London........ 326
Os pedis, removal of........ 315
Osseous deformities in the lower animals........ 173
Osler, William.—Extensive cystic disease in the pig........ 313
Ostrich breeding........ 81
Ostriches, diseases and accidents among........ 74

Parasites in American pork........ 254
stock........ 83
Parasiticide, a new........ 320
Parmly, Ehrick.—Abortion in cows........ 109
Pasteur, tribute to........ 326
and Koch........ 174
on rabies........ 178
Penis, amputation of........ 320
Pepsin of sheep, the........ 73
Personal........ 326

Pets, domestic, and skin disease.... 55
Philadelphia veterinarians and dishonest druggists.... 81
Physical diagnosis....11, 205, 275
Phitisis conveyed from dogs to man.... 180
Pigs, overfed.... 82
Pilocarpin, the action of, upon Horses.... 56
Pleuro-pneumonia contagiosa.... 243
Pneumothorax in a coati.... 321
Poisoning by arsensic.... 82
blood, in the lower vertebrates.... 329
Poll-evil, treatment of.... 323
Porter, William Henry, acute parenchymatous degeneration of the kidneys.... 87
Poultry, a good medicine for.... 73
Presentation....254, 259
Prophylactic Vaccine for "Rouget".... 175

Quarantine stations, cattle.... 83

Quarantine, the New York cattle.... 308

Quitters, Treatment of.... 323

Rabies, incubation of, in dogs.... 75

Rabies, recovery from.... 76

Ramsdell, E. Benjamin, M.D.—Physical Diagnosis....11, 205, 275

Rary system, improvement on the.... 262

Ration of hay for horse.... 81

Red water in cattle.... 255

Reports of Commencements and Societies....142, 231

Reviews....65, 165, 245, 318

Robertson, Dr.—Simple Lameness.... 240

Case of Jaundice.... 240

Rutherford, James.—Idiopathic tetanus.... 152

Sanders' Mission....230, 309
Sarcoma in a common fowl.... 321
Scabies from sarcoptes canis, incipient stages of.... 239
Scarlatina among horses.... 250
Sheep fluke.... 171
Shoeing a kicking horse or mule.... 327
Small-pox in birds.... 180
Snake, hyberuating, examination of.... 253
Sterescope in the study of anatomy.... 332
Stickler, Joseph W., M.D—Incipient stages of scabies from sarcoptes canis supra-pubic cystomy.... 213
Stomach digestion in the horse.... 53
Strychnine poisoning in a dog.... 235
Sulphur, use of.... 235
Swine plague.... 171
Syphilitic, inoculation immunity of animals, from.... 252

Tail, rubbing the ........ 331
Teeth of the horse and their diseases ........ 121
Test for iodoform ........ 172
Tetanus idiopathic ........ 61, 152
or traumatic ........ 151
Tintura iodoformi comp. ........ 172
Townsend, N. S., M.D —Milk sickness ........ 117
Transmission of tubercle from the human species to the domestic cat ... 249
Treasury cattle commission, report of ........ 181
Trichiasis—ingrowing eyelashes ........ 236
Trichina ........ 261
cold kills ........ 180
Trichinosis from eating horse flesh ........ 327
Tsetse fly ........ 81
Tubercle—bacillus in hens ........ 310
Tuberculosis and hydrothorax in a female rhinoceros ........ 249
Tumor cerebral ........ 262
Tumors, recurring cystic of the supra-scapular region ........ 157
Tympanitis terminating in rupture of stomach ........ 315
Typhoid fever among live stock ........ 84

Vaccination ........ 260
Venom copperhead ........ 260
Veterinarians, more needed ........ 256
specimen ........ 85
Veterinary Association, Ontario ........ 147
State ........ 311
College, American ........ 145
Columbia ........ 229
annual commencement of ........ 142
North Western ........ 81
Ontario ........ 231
one stool ........ 139
Colleges, English, and their graduates ........ 50
in New York city, the legal status of ........ 52
Congress international ........ 83
Convention, Illinois State ........ 259
hospital ........ 258
journal, a Western ........ 139
medical schools, Berlin ........ 81
news, English ........ 80
profession at large, to the ........ 140
school, Harvard ........ 212
new ........ 180
society, Ontario ........ 181
societies ........ 50
surgeons, State Convention of, in Chicago ........ 233
number of, in this country ........ 180
U. S. and the R. C. V. S ........ 307

Victims to wolves ........................................ 326
Villous membrane of stomach, thickening of........................... 325
Virchows health, Professor.......................................... 254
Virus, equine scarlatinal as a prophylactic against human scarlatina. . . 174
Vivisection question, the............................................. 182
Walley, Thomas, contraction of the foot in the horse..................... 273
Walton, Frank V., acute parenchymatous degeneration of the kidneys
in a mare........................................ 156
adenoma of the breast in a dog............. 59
fracture of the internal metatarsal......... 162
fracture of the os inorminata.............. 164
hamaturia associated with nephritis....... 161
intestinal calculus lodged in colon.......... 160
intestinal calculus from a horse............ 242
Wiltshire, Alfred.—The comparative physiology of menstruation....... 284.
Windolph, J. S.—Chronic rheumatism, arthritis with osteo-phatic outgrowths occurring in a young ox................................ 163
What is cruelty to a cat?............................................ 82
What shall he do with them?......................................... 328
Wound, penetrating, of abdomen—recovery.......................... 159

